# Austin-Type Meroterpenoids from Fungi Reported in the Last Five Decades: A Review

**DOI:** 10.3390/jof10020162

**Published:** 2024-02-19

**Authors:** Jia-Li He, Chang-Jing Chen, Yong-Hong Liu, Cheng-Hai Gao, Rui-Ping Wang, Wen-Fei Zhang, Meng Bai

**Affiliations:** 1 Ministry of Education Key Laboratory for Ecology of Tropical Islands, College of Life Sciences, Hainan Normal University, Haikou 571158, China; 13787315411@163.com (J.-L.H.); 18845166250@163.com (C.-J.C.); wrp@hainnu.edu.cn (R.-P.W.); 2 Guangxi Key Laboratory of Marine Drugs, Institute of Marine Drugs, Guangxi University of Chinese Medicine, Nanning 530200, China; yonghongliu@scsio.ac.cn (Y.-H.L.); gaoch@gxtcmu.edu.cn (C.-H.G.)

**Keywords:** fungi, austin-type meroterpenoids, structural diversity, biological activity

## Abstract

Austin was first isolated as a novel polyisoprenoid mycotoxin from *Aspergillus ustus* in 1976. Subsequently, some new austin-type meroterpenoids (ATMTs) have been continually found. This review attempts to give a comprehensive summary of progress on the isolation, chemical structural features, biological activities, and fungal biodiversity of 104 novel ATMTs from 5 genera of terrestrial- and marine-derived fungi reported from October 1976 to January 2023. The genera of *Penicillium* and *Aspergillus* are the two dominant producers, producing 63.5% and 30.8% of ATMTs, respectively. Moreover, about 26.9% of ATMTs display various pronounced bioactivities, including insecticidal, anti-inflammatory, cytotoxicity, antibacterial, and PTP1B inhibitory activities. The chemical diversity and potential activities of these novel fungal ATMTs are reviewed for a better understanding, and a relevant summary focusing on the source fungi and their taxonomy is provided to shed light on the future development and research of austin-type meroterpenoids.

## 1. Introduction

Microbial secondary metabolites differ from primary nutrients in that they are not essential for growth, but they play a vital role in the survival and adaptation of microbes in nature [1]. Fungi attract much attention from chemists and biologists due to the production of secondary metabolites with diverse structural skeletons and interesting bioactivities. Austin-type meroterpenoids (ATMTs) are a family of hybrid natural products with high diversity of intriguing scaffolds, but only 104 ATMTs have been characterized, which are a relatively rare branch of the terpenoid family and have often been isolated from fungi, especially from the genera *Penicillium* and *Aspergillus* [2,3,4,5,6,7,8]. The naturally occurring meroterpenoids derived from 3,5-dimethylorsellinic acid (DMOA) incorporated with farnesyl pyrophosphate (FPP) are the most common subclass [9,10,11,12,13], of which ATMTs are a class of special and important constituents. Based on the number of rings of the initial meroterpenoid in their biosynthesis pathway, ATMTs are classified into four categories, including tetracyclic, pentacyclic, hexacyclic, and heptacyclic systems. Interestingly, different types of ATMTs display broad and impressive biological activity [14,15,16], including insecticidal, antiphlogistic, antimicrobial, and antineoplastic effects, etc.

To date, no individual and comprehensive identification of the chemical structures of ATMTs has been reported. Therefore, this review was prepared to provide an overall coverage of the chemical constituents of the ATMTs reported in the last five decades (from October 1976 to January 2023) originating from fungi according to a classification of their chemical skeletons (two databases were used for the search: SciFinder and Web of Science). This review will provide information on the isolation, chemical structural features, biological activities, and fungal biodiversity of ATMTs, which will facilitate further research and exploitation of these structures. In this review, the biosynthesis pathways of these ATMTs are not discussed as they have been extensively reviewed by Wang, Abe, and Brakhage [17,18,19,20].

## 2. Austin-Type Meroterpenoids Compounds

### 2.1. Tetracyclic Systems Austin-Type Meroterpenoids

#### 2.1.1. Tetracyclic Systems-Rings A and B Are Spirocyclic

**Figure 1:**
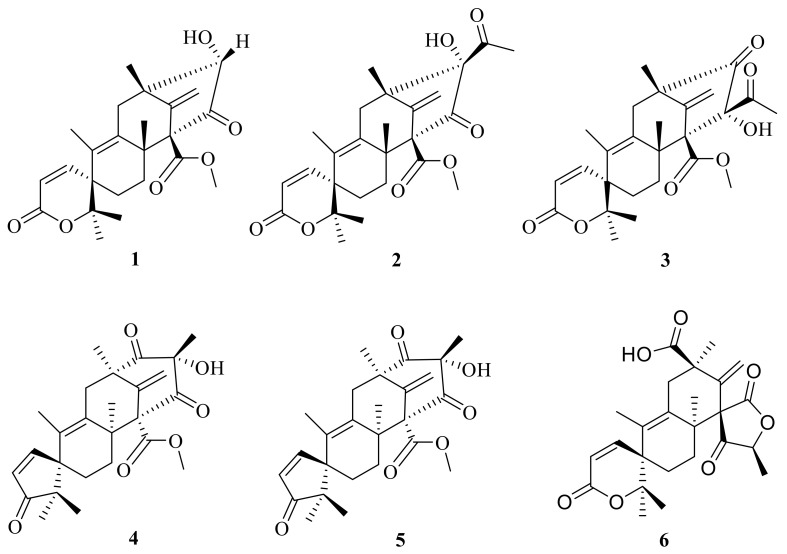
Chemical structures of compounds **1**–**6**.

This subgroup of ATMTs is characterized by a tetracyclic spirocyclic systems with six members (**1**–**6**) (Figure 1), including austinoneol (**1**) that was obtained from a *Penicillium* sp. [21] that was isolated from the root bark of *Melia azedarach* after surface sterilization and cultivated for three weeks on sterilized rice. The filamentous fungus *Aspergillus nidulans* has previously been found to produce two meroterpenoids, austinol and dehydroaustinol. The use of targeted deletions revealed that two separate gene clusters are required for meroterpenoid biosynthesis. One is a cluster of four genes including the polyketide synthase gene, *aus*A. The second is a cluster of 10 additional genes, including the prenyltransferase gene, *aus*N, located on a separate chromosome. Chemical analysis of mutant extracts enabled isolation of preaustinoid A5 (**2**), preaustinoid A4 (**3**), and preaustinoid A3 (**4**), which are either intermediates or shunt products from the biosynthetic pathway [17] (Figure 1). The filamentous fungus *Aspergillus oryzae* NSAR1 is used as part of a heterologous fungal expression system; *Aspergillus oryzae* NSAR1 is a quadruple auxotrophic mutant strain (*nia*D^−^, *s*C^−^, *Δarg*B, *ade*A^−^) that is used to produce austinoid C (**5**) [18]. Furthermore, one unusual austin-type meroterpenoid is penicianstinoid C (**6**), which was the first ATMT with a unique 6/6/6/5 rearranged tetracyclic skeleton possessing two unusual spirocyclic moieties (2-oxaspiro[5.5]undeca-4,7-dien-3-one and 6-methylene-2-oxaspiro[4.5]decane-1,4-dione); it was obtained from the mangrove-derived fungus *Penicillium* sp. TGM112. In addition, this compound shows inhibitory activity against newly hatched larvae of *Helicoverpa armigera* Hubner (IC_50_ = 100 μg/mL) [22].

#### 2.1.2. Tetracyclic Systems-Rings A and B Are Bicyclic Fused

**Figure 2:**
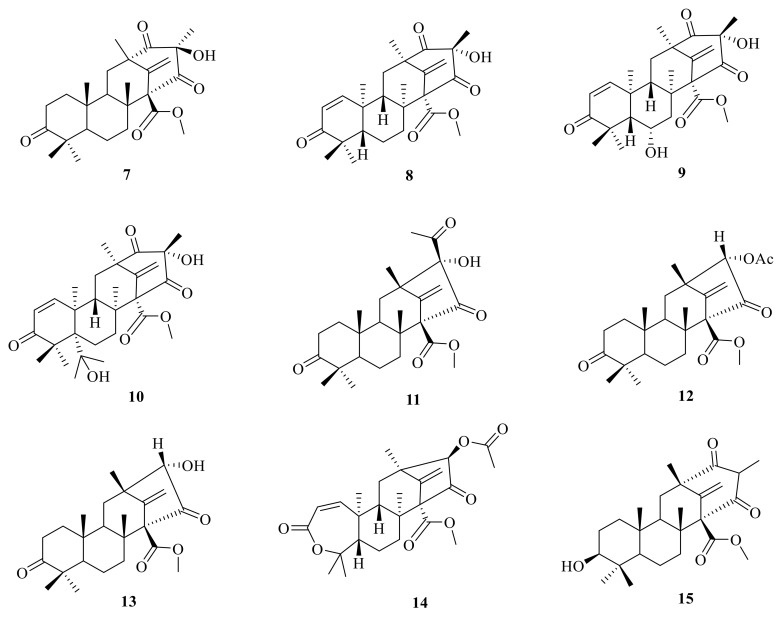
Chemical structures of compounds **7**–**15**.

An endophytic fungus *Penicillium* sp. is cultured from solid rice medium and was isolated from the root bark of *Melia azedarach*. It produced two new tetracyclic system ATMTs, preaustiniod A (**7**) and B (**11**), and they exhibited moderate bacteriostatic effects on *Escherichia coli*, *Staphylococcus aureus*, *Pseudomonas aeruginosa*, and *Bacillus* sp. [23] (Figure 2). Genome mining of the fungus *Aspergillus oryzae* NSAR1 led to the isolation of three tetracyclic system ATMTs, preaustinoid C (**8**), 5-hydroxyberkeleyone A (**16**), and berkeleyone A (**17**) [18]. By employing a large-scale culture approach, asperanstinoid E (**9**) was obtained from the fungus *Aspergillus calidoustus*, which was isolated from wetland soil collected at Dianchi Lake [24]. Penicianstinoid D (**10**), an unusual austin-type meroterpenoid with a 6/6/6/6 tetracyclic skeleton containing an octahydro-2H-chromen-2-one unit, was obtained from *Penicillium* sp. TGM112 [23]. The endophytic fungus *Penicillium* sp. derived from the root of *Panax notoginseng* yielded three new ATMTs, including preaustinoid B1 (**12**), preaustiniod A1 (**18**), and A2 (**19**) [25] (Figure 2). Two additional meroterpenes were isolated and identified from rice cultures of *Penicillium* sp., a fungus obtained from the root bark of *Melia azedarach*. These new compounds were named preaustinoid B2 (**13**), and preaustinoid A3 (**4**) [26]. The undescribed meroterpenoid preaustinoid C (**14**) was isolated from *Penicillium* sp. RO-11, which was collected from the sediments of a hydrothermal spring located in the southwestern area of Saudi Arabia. Preaustinoid C showed significant activity against lipopolysaccharide (LPS)-induced NO production and a selective effect on IL-2 and IFN*-γ* gene regulation in activated Jurkat cells [27]. The mutant filamentous fungus *Aspergillus nidulans* has been found to produce protoaustinoid A (**15**) [17].

In addition, peniscmeroterpenoid N (**20**) was isolated from the marine-derived fungus *Penicillium sclerotiorum* GZU-XW03-2 [28], which was obtained from the intestinal tract of an *Onchidium* sp. collected from Xuwen in Guangdong province, China. The fungus *Penicillium purpurogenum* obtained from rotting fruit of the tree *Averrhoa bilimbi* growing in Sri Lanka yielded four new meroterpenoids, named dhilirolides F–I (**21**–**24**) [12]. In addition, 3,16-epoxy-preaustinoid D (**25**) is a rare austin meroterpenoid analogue with an open A ring that also features an undescribed oxygen bridge between C-3 and C-16 to construct an unexpected tetrahydrofuran ring; this was isolated and characterized from the fungus *Aspergillus calidoustus* [29].

**Figure 3:**
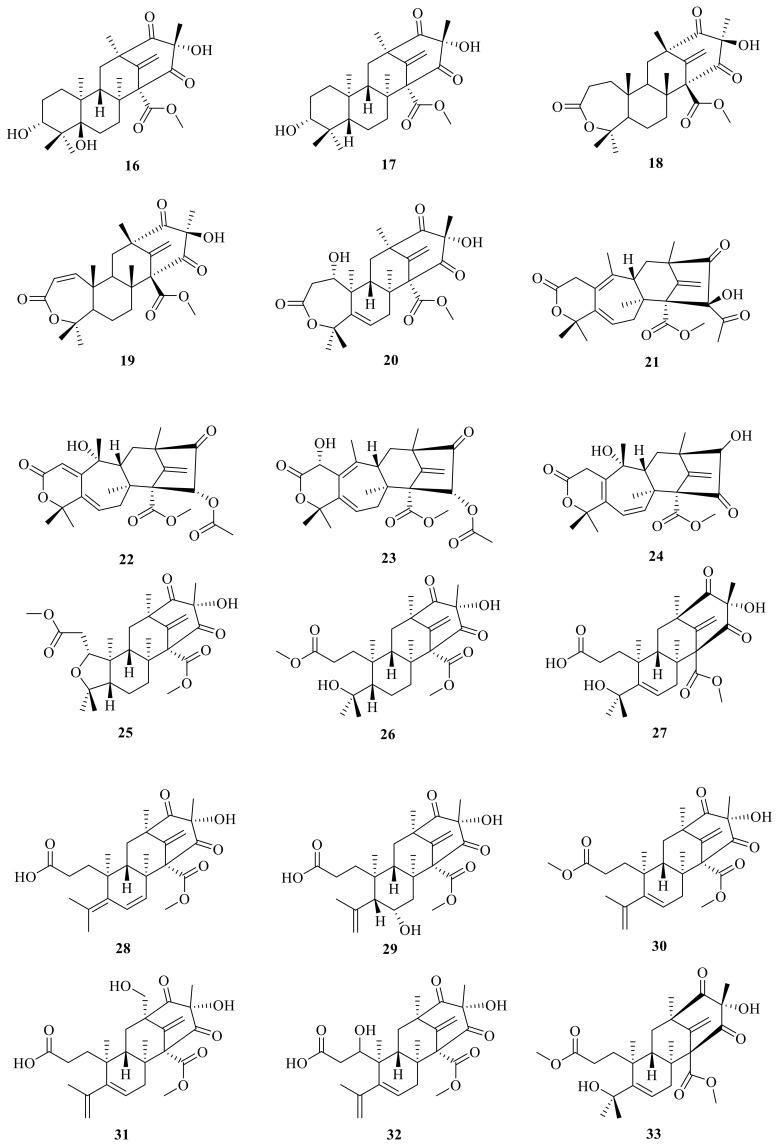
Chemical structures of compounds **16**–**33**.

Furthermore, two new meroterpenoids, namely, 1-methoxy-hydropreaustinoid A1 (**26**) and hydroberkeleyone B (**27**), have been isolated through the aid of Liquid Chromatograph Mass Spectrometer (LC-MS) from the sponge-derived fungus *Eupenicillium* sp. 6A-9, and both of them have immune-suppressive activities with IC_50_ values of 42.3 and 28.5 μM, respectively [30]. Peniscmeroterpenoids F (**28**) and G (**29**) were also isolated from the marine-derived fungus *Penicillium sclerotiorum* GZU-XW03-2 [31]. SSW03M2 GY was isolated (4*S*, 5*S*, 7*R*, 9*S*, 11*R*, 12*S*)-1-methoxyberkeleyone C (**30**) from a *Penicillium* sp. that was derived from sediment at Seosan bay, South Korea; compound **30** showed anti-virulence activity by significantly inhibiting α-toxin (Hla) secreted by methicillin-resistant *Staphylococcus aureus* without growth inhibition at 10 μg/mL [32]. Peniscmeroterpenoids K–M (**31**–**33**) were also isolated from the marine-derived fungus *Penicillium sclerotiorum* GZU-XW03-2. Specifically, peniscmeroterpenoid K (**31**) was the first isolate where the C-24 was oxidized, and peniscmeroterpenoid M (**33**) (Figure 3) inhibited the production of nitric oxide (NO) in RAW264.7 cells with an IC_50_ value of 48.04 ± 2.51 μM [28].

**Figure 4:**
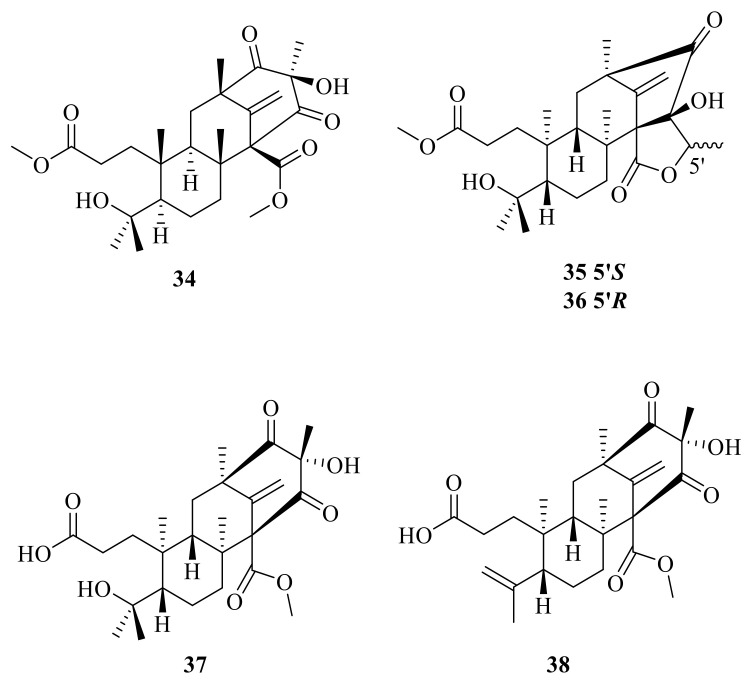
Chemical structures of compounds **34**–**38**.

Preaustinoid D (**34**) was isolated from the endophytic fungus *Penicillium* sp. T2-8 associated with *Gastrodia elata* and showed moderate activity against *Candida albicans* with an MIC of 128 μg/mL [33]. Bioinformatics analysis of the gene clusters in association with the qRT-PCR detection revealed the amplification of two key genes (clusters A and B) for the sponge-associated fungus *Penicillium brasilianum* WZXY-m122-9. Chromatographic separation of the EtOAc extract from the large-scale fermentation of this fungal strain resulted in the isolation of two new ATMTs, namely, brasilianoids D and E (**35**–**36**) [34]. The marine-derived fungal isolate *Penicillium* sp. SF-5497 resulted in the isolation of two new meroterpenoids, named preaustinoid A6 (**37**) and preaustinoid A7 (**38**); furthermore, preaustinoid A6 (**37**) (Figure 4) inhibited protein tyrosine phosphatase-1B (PTP1B) in a noncompetitive manner, with a K_i_ value of 17.0 µM [35].

### 2.2. Pentacyclic Systems Austin-Type Meroterpenoids

#### 2.2.1. Pentacyclic Systems-Rings A and B Are Spirocyclic—Typical Austin-Type Meroterpenoids

**Figure 5:**
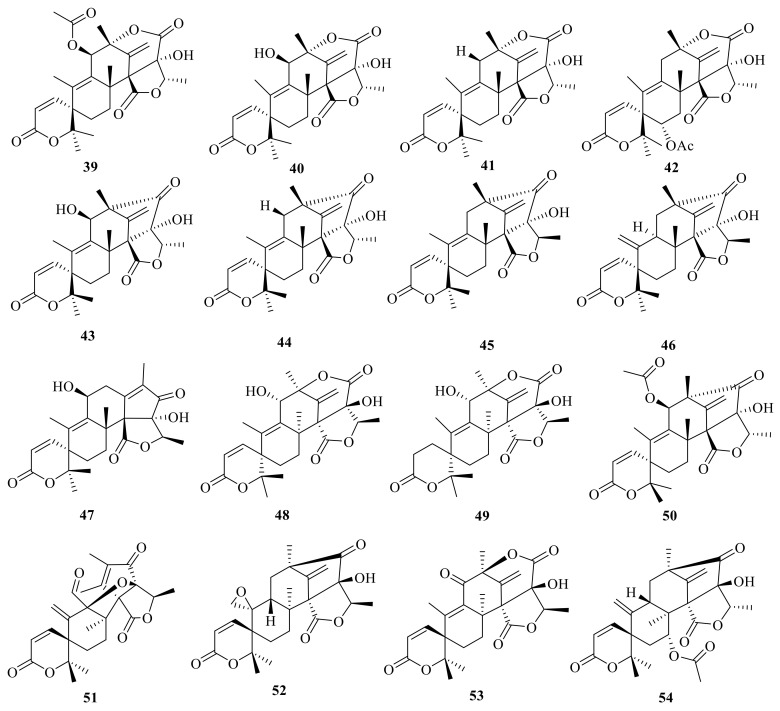
Chemical structures of compounds **39**–**54**.

Austin (**39**) (Figure 5) is a novel ATMT isolated from a strain of *Aspergillus ustus* that was found on stored black-eyed peas (*Vigna sinensis*). Austin (**39**) showed the gross signs of toxicity in cockerels, such as a general listlessness followed either by eventual improvement (250 mg/kg, oral) or ataxia and death (375 mg/kg, oral) [3] (Figure 5). Austinol (**40**) and isoaustin (**42)** were isolated from an *A. ustus* strain [7]. Austinolide (**41**) and isoaustinone (**44**) are additional ATMTs produced by the rice cultures of *Penicillium* sp. [26]. Moreover, chemical examination of *Aspergillus nidulans* resulted the isolation of 11 *β*-hydroxyisoaustinone (**43**), (5′*R*)-isoaustinone (**45**), and neosuatinone (**47**) [17].

Neoaustin (**46**) was obtained from a soil isolate of *Penicillium* sp. MG-11 [36]. Moreover, ED-2 (**48**) was isolated from a strain of *Emericella nidulans* var. *dentata*, and ED-2 (**48**) was hydrogenated with H_2_/PtO_2_ and afforded the crystalline dihydro-derivative ED-2H (**49**) [6]. In addition, 11β-acetoxyisoaustinone (**50**) was isolated from the seagrass-derived fungus *Pestalotiopsis* sp. PSU-ES194 [37]. In the same year, 11β-acetoxyisoaustinone (**50**) was also isolated from *Penicillium* sp., an endophytic fungus obtained from *Dysosma versipellis* [38]. Bioinformatics analysis of the synthetic gene clusters in association with the qRT-PCR detection of the marine-derived *Penicillium brasilianum* WZXY-m122-9 isolate yielded four new ATMTs, namely brasilianoids G–I (**51**–**53**) and brasilianoid L (**54**) (Figure 5). Compound **54** showed cytotoxicity activity against RAW264.7, IEC-6, and A549 with IC_50_ values of 84.67, 2.52 and 180.5 μg/mL, respectively [1].

**Figure 6:**
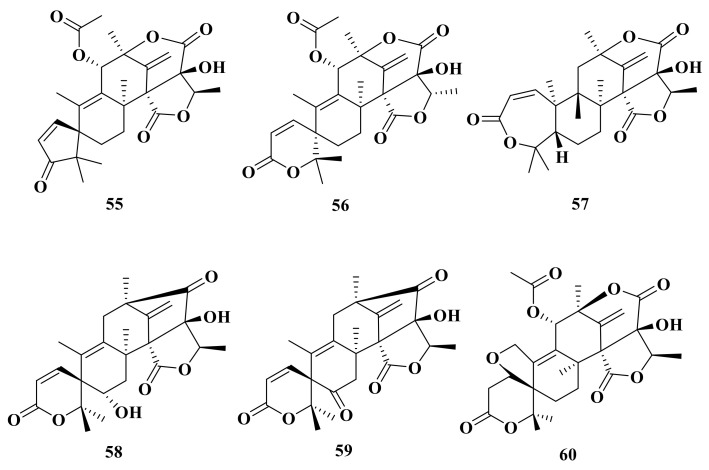
Chemical structures of compounds **55**–**60**.

Moreover, asperaustins A–C (**55**–**57**) were isolated from a marine-derived *Aspergillus* sp. Compound **55** possesses an unusual spiro[4.5]deca-3,6-dien-2-one moiety with a unique 5/6/6/6/5 pentacyclic skeleton [2] (Figure 6). Under the guidance of MS/MS-based molecular networking, 6-hydroxyisoaustinone (**58**) and 6-ketoisoaustinone (**59**) were isolated from the *Penicillium* sp. GDGJ-285 [39]. Penicianstinoid E (**60**), an unusual austin-type meroterpenoid that possesses a 6/5/6/6/6/5 fused hexacyclic skeleton with an uncommon five-membered ether ring system, was obtained from *Penicillium* sp. TGM112. Compound **60** showed inhibitory activity against newly hatched larvae of *Helicoverpa armigera* Hubner with an IC_50_ value of 200 μg/mL [22]. Asperanstinoid A was obtained from the fungus *A. calidoustus* and has the same structure as penicianstinoid E (**60**) [24].

#### 2.2.2. Pentacyclic Systems-Rings A and B Are Bicyclic Fused

**Figure 7:**
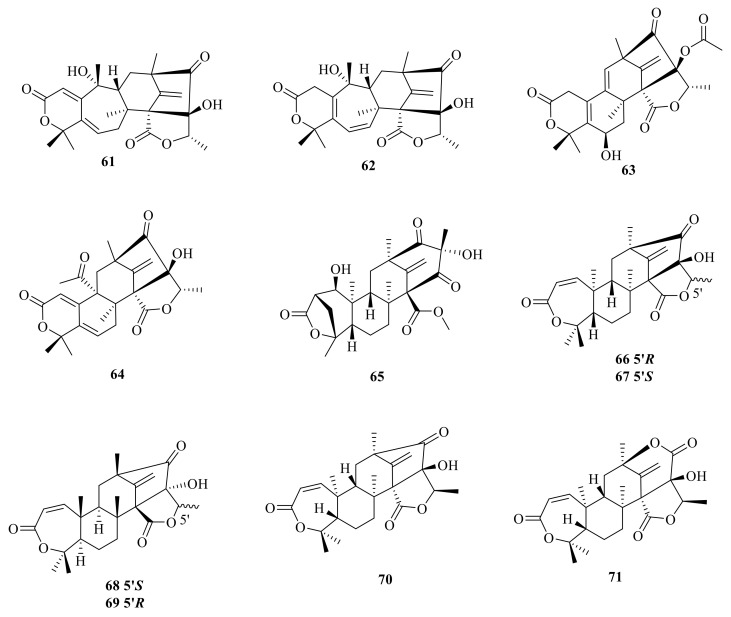
Chemical structures of compounds **61**–**71**.

Dhilirolide D (**61**), a member of the family of secondary metabolites with a putative meroterpenoid biogenetic origin and unprecedented dhilirane and isodhilirane carbon skeletons, was isolated from a laboratory culture of *P. purpurogenum* collected in Sri Lanka [13] (Figure 7). In addition, continued chemical investigation of laboratory cultures of *P. purpurogenum* yielded three new ATMTs, named dhilirolide E (**62**), dhilirolide K (**63**), and dhilirolide M (**64**) [12].

Chromatographic separation of the EtOAc extract of the large-scale fermentation of the sponge-associated *P. brasilianum* WZXY-m122-9 resulted in the isolation of three new ATMTs named brasilianoids A–C (**65**–**67**). Compound **65** showed significantly stimulated the expression of filaggrin and caspase-14 in HaCaT cells in dose-dependent manner; moreover, compounds **66** and **67** showed moderate inhibition against NO production in LPS-induced RAW 264.7 macrophages with IC_50_ values of 37.69 ± 5.25 μM and 33.76 ± 3.13 μM, respectively [34] (Figure 7). Preaustinoids E–F (**68**–**69**) were isolated from the culture broth of a *Penicillium* sp. fungus collected from Chuja-do, Korea [40]. Asperaustin C (**70**) was isolated from a marine-derived *Aspergillus* sp. [2]. In addition, a new ATMT brasilianoid K (**71**) was isolated from the marine-derived fungus *P. brasilianum* WZXY-m122-9 [1].

### 2.3. Hexacyclic Systems Austin-Type Meroterpenoids

#### 2.3.1. Hexacyclic Systems-Rings A and B Are Spirocyclic

**Figure 8:**
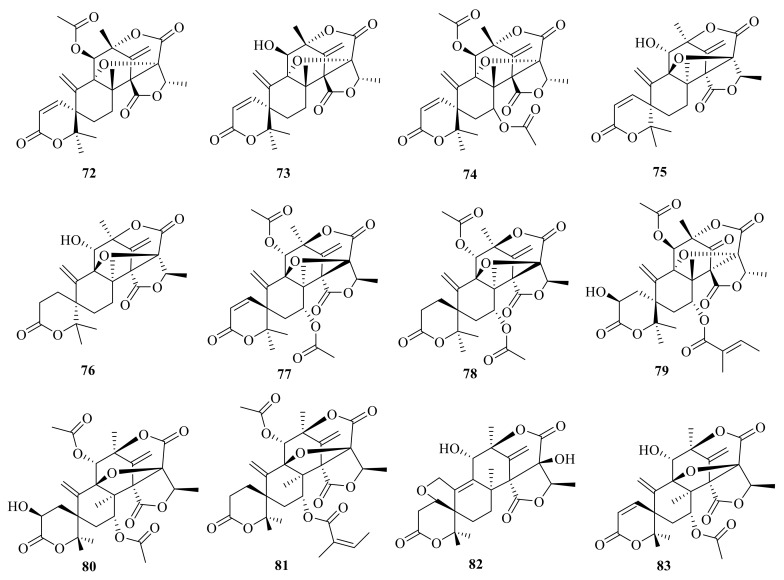
Chemical structures of compounds **72**–**83**.

A new ATMT isoaustinone (**72**) was isolated from a strain of *Aspergillus variecolor*, and it inhibited the growth of newly third-instar larvae of *Aedes aegypti* with an LC_50_ value of 2.9 ppm [7] (Figure 8). Dehydroaustin (**73**) and dehydroaustinol (**74**) were isolated from a soil fungus of *Penicillium* sp. MG-11. Compound **74** showed antimicrobial activity against *Escherichia coli* with an MIC value of 250 μg/mL and inhibited the growth of newly third-instar larvae of *Aedes aegypti* with an LC_50_ value of 7.3 ppm [36]. ED-1 (**75**) was isolated from a strain of *Aspergillus ustus*, and ED-1 (**75**) was hydrogenated with H_2_/PtO_2_ and afforded a crystalline dihydro derivative ED-1H (**76**) [41]. Acetoxydehydroaustin B′ (**77**) and 1,2-dihydro-acetoxydehydroaustin B′ (**78**) were isolated as a mixed crystal from the mangrove endophytic fungus *Aspergillus* sp. 085241B [42]. Furthermore, PF1364 (**79**) was isolated from *Aspergillus* sp. PF1364 and showed the control of pests and insects, such as the greenhouse whitefly [43].

2-Hydroacetoxydehydroaustin (**80**) was isolated from the mangrove endophytic fungus *Aspergillus* sp. 16-5c [44] (Figure 8). In addition, 1,2-dehydro-terredehydroaustin (**81**) was isolated from the mangrove endophytic fungus *Aspergillus terreus* and inhibited nitric oxide (NO) production with an IC_50_ value of 42.3 μM [45]. Furanoaustinol (**82**) and 7-acetoxydehydroaustinol (**83**) (Figure 8) were isolated from the ethyl acetate extract of the marine-derived fungal strain *Penicillium* sp. SF-5497. Compound **82** weakly inhibited the activity of protein tyrosine phosphatase 1B in a dose-dependent manner with an IC_50_ value of 77.2 μM. In addition, compound **83** weakly suppressed the overproduction of nitric oxide in lipopolysaccharide-challenged BV2 microglial cells with an IC_50_ value of 61.0 μM [8].

**Figure 9:**
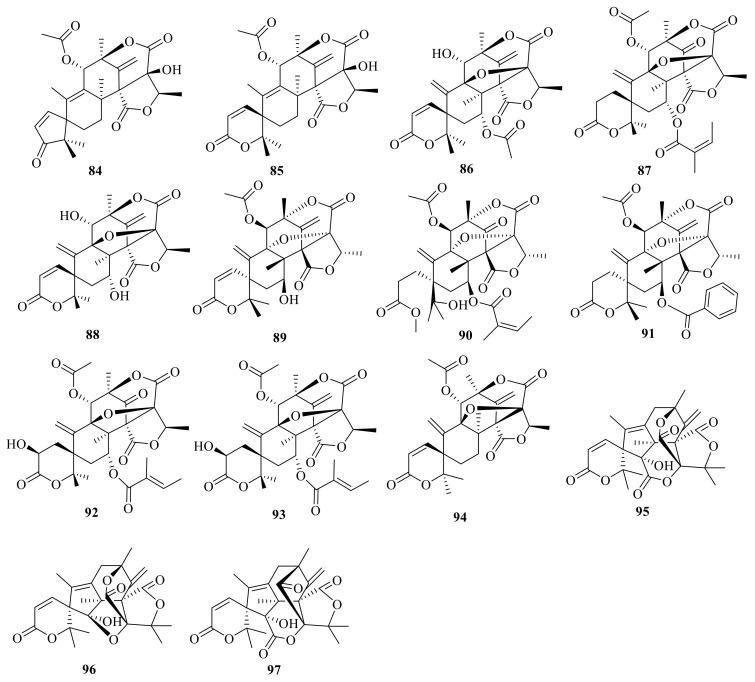
Chemical structures of compounds **84**–**97**.

Asperaustins A–B (**84**–**85**) (Figure 9) were isolated from a marine-derived *Aspergillus* sp. [2]. Brasilianoid J (**86**) was isolated from the marine-derived fungus *P. brasilianum* WZXY-m122-9 [27] (Figure 9). Penicianstinoids A–B (**87**-**88**) were obtained from the mangrove-derived fungus *Penicillium* sp. TGM112, and compounds **87** and **88** showed growth inhibition activity against newly hatched larvae of *Helicoverpa armigera* Hubner with IC_50_ values of 200 μg/mL. Compounds **87** and **88** displayed activity against *Caenorhabditis elegans* with EC_50_ values ranging from 9.4 ± 1.0 μg/mL to 38.2 ± 0.6 μg/mL [15]. In addition, 7-hydroxydehydroaustin (**89**) was isolated from the seagrass-derived fungus *Pestalotiopsis* sp. PSU-ES194 [37]. Austinone (**90**) was isolated from *Penicillium* sp. Y-5-2 [46].

Ustusaustin A (**91**) was isolated and identified from a culture extract of *Aspergillus ustus* TK-5, which obtained from the inner tissue of the ascidian *Pyuramomus* [47]. Three 3,5-dimethylorsellinic acid (DMOA)-based meroterpenoids, namely asperanstinoids B–D (**92**–**94**) (Figure 9), were obtained from the fungus *Aspergillus calidoustus*, which was isolated from wetland soil collected at Dianchi Lake [24]. Under the guidance of MS/MS-based molecular networking, three novel skeleton meroterpenoids, peniclactones A–C (**95**–**97**) (Figure 9), were isolated from *Penicillium* sp. GDGJ-285 [39]. Peniclactones A–C (**95**–**97**) represent the first meroterpenoids featuring a 6/5/6/6/5/6 (**95**), 6/5/6/6/5/5 (**96**), and 6/5/6/5/5/6 (**97**) hexacyclic ring systems, respectively. Bioassays showed that **97** inhibited nitric oxide production in lipopolysaccharide-induced RAW 264.7 macrophage cells with an IC_50_ value of 39.03 μM.

#### 2.3.2. Hexacyclic Systems-Rings A and B Are Bicyclic Fused

**Figure 10:**
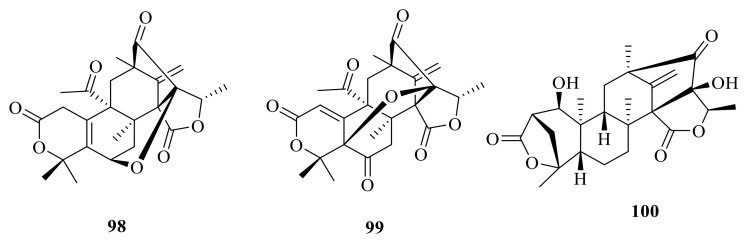
Chemical structures of compounds **98**–**100**.

Dhilirolide L (**98**) and dhilirolide N (**99**) were isolated from *P. purpurogenum*, which was obtained from rotting fruit of a *Averrhoa bilimbi* tree growing in Sri Lanka. Compound **98** showed significant feeding inhibition and sublethal developmental disruption in the cabbage looper *Trichoplusiani* with a DC_50_ value of 5.9 μg/cm^2^ [12] (Figure 10). Brasilianoid F (**100**) was obtained from the large-scale fermentation of *P. brasilianum* WZXY-m122-9 [34].

### 2.4. Heptacyclic Systems Austin-Type Meroterpenoids

#### Heptacyclic Systems-Rings A and B Are Bicyclic Fused

**Figure 11:**
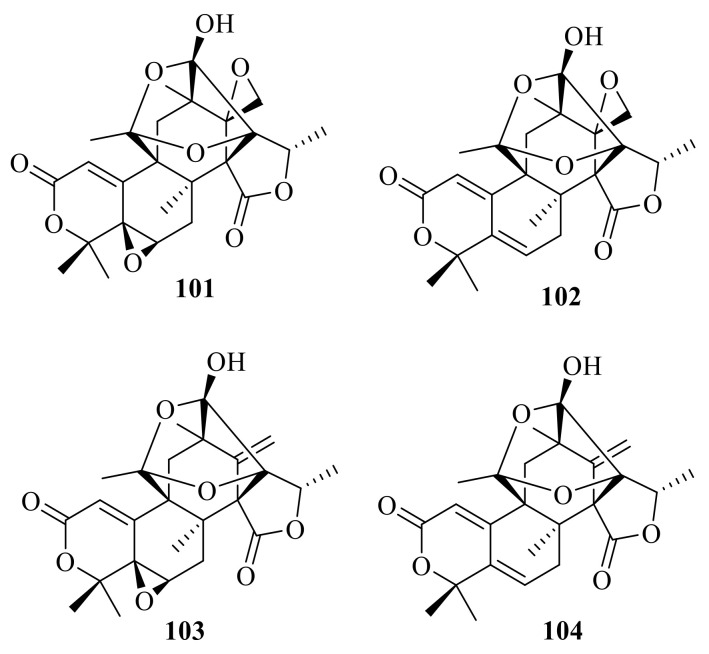
Chemical structures of compounds **101**–**104**.

Dhilirolides A–C (**101**–**103**) were isolated from laboratory cultures of the fruit-infecting fungus *P. purpurogenum* collected in Sri Lanka [13] (Figure 11). The fungus *P. purpurogenum* obtained from rotting fruit of a *A. bilimbi* tree growing in Sri Lanka yielded one new austin-type meroterpenoid, named dhilirolide J (**104**) [25].

## 3. Comprehensive Overview and Conclusions

**Figure 12:**
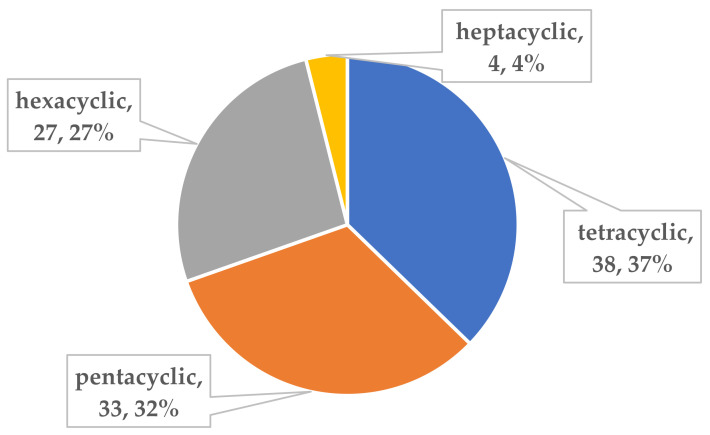
Classification of all ATMTs compounds.

**Figure 13:**
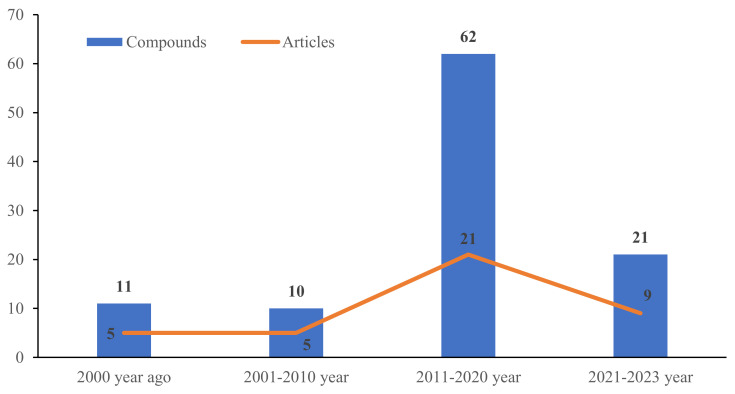
The production of austin-type meroterpenoids and published articles per decade.

**Figure 14:**
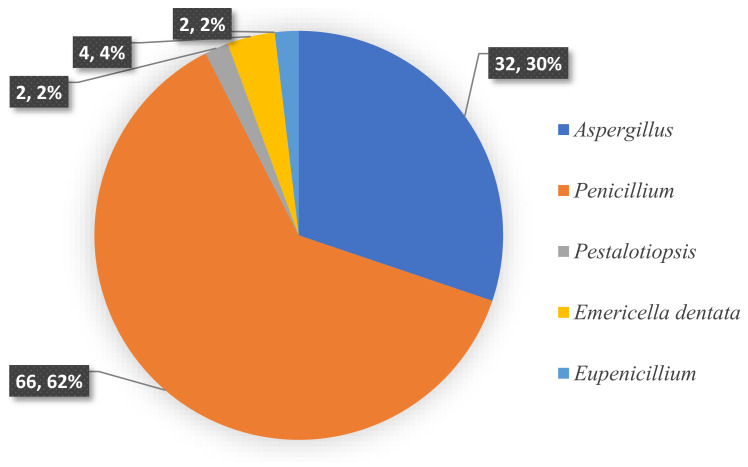
Fungal species distribution of isolated austin-type meroterpenoids.

**Figure 15:**
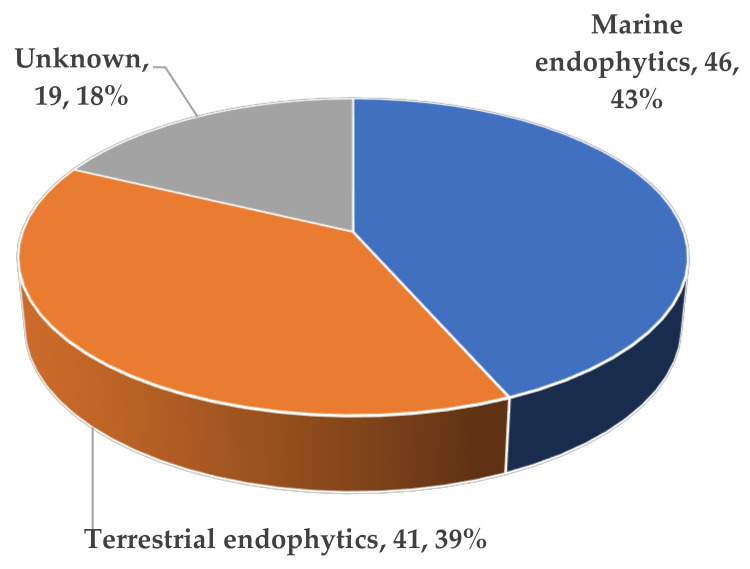
Fungal producers of austin-type meroterpenoids.

To the best of our knowledge, investigations on the chemical constituents of ATMTs in the last five decades have reported fungal biodiversity, and total of 104 novel ATMTs from 5 genera of terrestrial- and marine-derived fungi have been reported in 40 research papers published from October 1976 to January 2023 (Table 1, Figure 12, Figure 13, Figure 14 and Figure 15). In total, 37% of the compounds are categorized as the tetracyclic type (up to 38 compounds) followed by pentacyclic (32%, 33), hexacyclic (27%, 29), and heptacyclic (4%, 4) types (Figure 12). This review summarizes the source, chemistry, and biological activities of the novel ATMTs. The majority of the ATMTs have been isolated since 2010, accounting for about 80% of all reported ATMTs (83/104) and 75% of the published articles (Figure 13). The increase was largely due to improvements in isolation procedures. All of the published ATMTs that have been isolated and identified in various filamentous fungi, most of them were produced by *Penicillium* (62%) and *Aspergillus* (30%), representing more than 90% of the secondary metabolites reported (Figure 14). The remaining (about 10%) were produced by *Pestalotiopsis* (2%), *Eupenicillium* (2%), and *Emericella* (4%).

**Figure 16:**
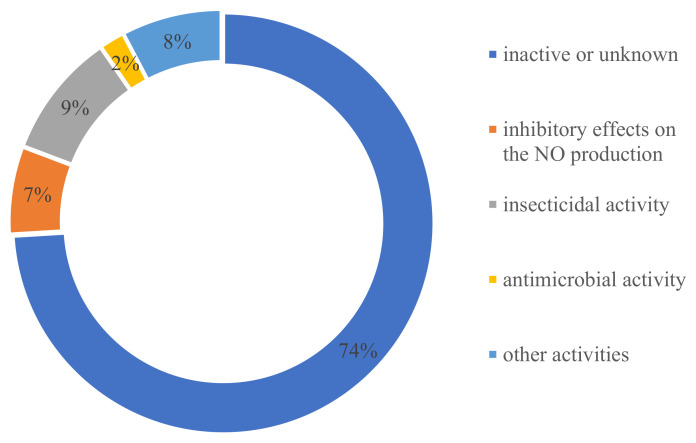
Bioactivity distribution of austin-type meroterpenoids.

In terms of the source distribution of compounds, one surprising discovery was that all of these novel ATMTs were isolated from fungi, including 39% terrestrial- and 43% marine-derived endophytic fungi, much more than those produced by unknown sources (18%) (Figure 15). The results showed that these kinds of ATMTs are a very special class of fungi metabolites, and more chemical ecological research needs to be carried out. Overall, this review provides a comprehensive overview of the diverse chemical structures and bioactive properties of 104 new ATMTs that have been isolated from fungi in the last five decades. Nearly 26% of the ATMTs showed bioactivities. About 7% exhibited inhibitory effects on NO production. Interestingly, about 9% ATMTs have been showed to possess selective insecticidal activity, about 2% exhibit antimicrobial activity, and 8% demonstrate other activities, including cytotoxicity and antityrosinase activity (Figure 16), revealing the untapped potential of activity ATMTs in pesticides and medicinal applications. However, for most of the isolated ATMTs, the lack of activity analysis and pharmacodynamic evaluation limit their application.

In summary, a total 104 of ATMTs have been isolated and characterized to date since the initial discovery of austin-type meroterpenoids in 1976. Although ATMTs have a distinct a unique chemical skeleton and potential biological activities, the unavailability of large amounts of natural austin-type meroterpenoids and purification challenges due to their structural complexity hindering efficient chemical synthesis have hindered further research. Most of the ATMTs with novel skeletons and biological activities have been discovered in recent years. The further development and application of these compounds is important, and the identification of promising lead compounds for the development of drugs is a critical future direction of study.

## Figures and Tables

**Table 1:** The compounds isolated from fungi (the structures of the compounds are illustrated in Figure 1, Figure 2, Figure 3, Figure 4, Figure 5, Figure 6, Figure 7, Figure 8, Figure 9, Figure 10 and Figure 11).

Compound	Molecular Formula	Producer Strain	Habitat	Activity	Ref
ustinoneol A (**1**)	C_24_H_30_O_6_	*Penicillium* sp.	the root bark of *Melia azedarach*	NA ^b^	[21]
preaustinoid A5 (**2**)	C_26_H_32_O_7_	*Aspergillus nidulans*	NR ^a^	NA	[17]
preaustinoid A4 (**3**)	C_26_H_32_O_7_	*Aspergillus nidulans*	NR	NA	[17]
preaustinoid A3 (**4**)	C_26_H_32_O_7_	*Aspergillus nidulans*	NR	NA	[17]
		*Penicillium* sp.	the root bark of *Melia azedarach*	NA	[26]
austinoid C (**5**)	C_26_H_32_O_6_	*Aspergillus oryzae* NSAR1	NR	NA	[18]
penicianstinoid C (**6**)	C_25_H_30_O_7_	*Penicillium* sp. TGM112	the mangrove *Brguiera sexangula* var. *rhynchopetala*	inhibitory activity against newly hatched larvae of *Helicoverpa armigera* Hubner (IC_50_ = 100 μg/mL);	[22]
preaustinoid A (**7**)	C_26_H_36_O_6_	*Penicillium* sp.	the root bark of *Melia azedarach*	NA	[23]
preaustinoid C (**8**)	C_26_H_34_O_6_	*Aspergillus oryzae* NSAR1	NR	NA	[18]
asperanstinoid E (**9**)	C_26_H_34_O_7_	*Aspergillus calidoustus*	Dianchi Lake	NA	[24]
penicianstinoid D (**10**)	C_29_H_40_O_7_	*Penicillium* sp. TGM112	the mangrove *Brguiera sexangula* var. *rhynchopetala*	NA	[22]
preaustinoid B (**11**)	C_26_H_36_O_6_	*Penicillium* sp.	the root bark of *Melia azedarach*	NA	[23]
preaustinoid B1 (**12**)	C_26_H_36_O_6_	*Penicillium* sp.	the root bark of *Melia azedarach*	NA	[25]
preaustinoid B2 (**13**)	C_24_H_34_O_5_	*Penicillium* sp.	the root bark of *Melia azedarach*	NA	[26]
preaustinoid C (**14**)	C_26_H_34_O_7_	*Penicillium* sp. RO-11	the sediments of a hydrothermal spring	significant activity against LPS-induced NO production	[27]
protoaustinoid A (**15**)	C_26_H_38_O_5_	*Aspergillus nidulans*	NR	NA	[17]
5-hydroxyberkeleyone A (**16**)	C_26_H_38_O_7_	*Aspergillus oryzae* NSAR1	NR	NA	[18]
berkeleyone A (**17**)	C_26_H_38_O_6_	*Aspergillus oryzae* NSAR1	NR	NA	[18]
preaustinoid A1 (**18**)	C_26_H_36_O_7_	*Penicillium* sp.	the root bark of *Melia azedarach*	NA	[25]
preaustinoid A2 (**19**)	C_26_H_34_O_7_	*Penicillium* sp.	the root bark of *Melia azedarach*	NA	[25]
peniscmeroterpenoid N (**20**)	C_26_H_34_O_8_	*Penicillium sclerotiorum* GZU-XW03–2	the intestinal tract of the *Onchidium* sp.	NA	[28]
dhilirolide F (**21**)	C_26_H_32_O_7_	*Penicillium purpurogenum*	*Averrhoa bilimbi* fruit	NA	[12]
dhilirolide G (**22**)	C_26_H_32_O_8_	*Penicillium purpurogenum*	*Averrhoa bilimbi* fruit	NA	[12]
dhilirolide H (**23**)	C_26_H_32_O_8_	*Penicillium purpurogenum*	*Averrhoa bilimbi* fruit	NA	[12]
dhilirolide I (**24**)	C_24_H_30_O_7_	*Penicillium purpurogenum*	*Averrhoa bilimbi* fruit	NA	[12]
3,16-epoxy-preaustinoid D (**25**)	C_26_H_36_O_8_	*Aspergillus calidoustus*	Dianchi Lake	NA	[29]
1-methoxy-hydropreaustinoid A1 (**26**)	C_27_H_40_O_8_	*Eupenicillium* sp. 6A-9	the marine sponge *Plakortis simplex*	the immune-suppressive activities (IC_50_ = 42.3 μM)	[30]
hydroberkeleyone B (**27**)	C_26_H_36_O_8_	*Eupenicillium* sp. 6A-9	the marine sponge *Plakortis simplex*	the immune-suppressive activities (IC_50_ = 28.5 μM)	[30]
peniscmeroterpenoid F (**28**)	C_26_H_34_O_7_	*Penicillium sclerotiorum* GZU-XW03-2	the intestinal tract of the *Onchidium* sp.	NA	[31]
peniscmeroterpenoid G (**29**)	C_26_H_36_O_8_	*Penicillium sclerotiorum* GZU-XW03-2	the intestinal tract of the *Onchidium* sp.	NA	[31]
(4*S*, 5*S*, 7*R*, 9*S*, 11*R*, 12*S*)-1-methoxyberkeleyone C (**30**)	C_27_H_36_O_7_	*Penicillium* sp. SSW03M2 GY	a sediment at Seosan bay	significantly inhibited the production of α-toxin (Hla) by greater than 85% (at 10 μg/mL)	[32]
peniscmeroterpenoid K (**31**)	C_26_H_34_O_8_	*Penicillium sclerotiorum* GZU-XW03–2	the intestinal tract of the *Onchidium* sp.	NA	[28]
peniscmeroterpenoid M (**32**)	C_26_H_34_O_8_	*Penicillium sclerotiorum* GZU-XW03–2	the intestinal tract of the *Onchidium* sp.	NA	[28]
peniscmeroterpenoid L (**33**)	C_27_H_38_O_8_	*Penicillium sclerotiorum* GZU-XW03–2	the intestinal tract of the *Onchidium* sp.	inhibitory effects on NO production (IC_50_ = 48.04 ± 2.51 μM)	[28]
preaustinoid D (**34**)	C_27_H_40_O_8_	*Penicillium* sp. T2-8	fresh rhizomes of *Gastrodia elata*	antimicrobial (*Candida albicans*: 128 μg/mL);	[33]
brasilianoid D (**35**)	C_26_H_38_O_7_	*Penicillium brasilianum* WZXY-m122-9	sponge	NA	[34]
brasilianoid E (**36**)	C_26_H_38_O_7_	*Penicillium brasilianum* WZXY-m122-9	sponge	NA	[34]
preaustinoid A6 (**37**)	C_26_H_38_O_8_	*Penicillium* sp. SF-5497	sea sand	inhibited PTP1B activity with a *K*_i_ value of 17.0 μM)	[35]
preaustinoid A7 (**38**)	C_26_H_36_O_7_	*Penicillium* sp. SF-5497	sea sand	NA	[35]
austin (**39**)	C_27_H_32_O_9_	*Aspergillus ustus*	black-eyed peas (*Vigna sinensis*)	gross signs of toxicity in cockerels (listlessness: 250 mg/kg; death: 375 mg/kg)	[3]
austinol (**40**)	C_25_H_30_O_8_	*Aspergillus ustus*	NR	NA	[7]
austinolide (**41**)	C_25_H_30_O_7_	*Penicillium* sp.	the root bark of *Melia azedarach*	NA	[26]
isoaustin (**42**)	C_27_H_32_O_9_	*Penicillium diversum*	NR	NA	[7]
11*β*-hydroxyisoaustinone (**43**)	C_25_H_30_O_7_	*Aspergillus nidulans*	NR	NA	[17]
isoaustinone (**44**)	C_25_H_30_O_6_	*Penicillium* sp.	the root bark of *Melia azedarach*	NA	[26]
(5′*R*)-isoaustinone (**45**)	C_25_H_30_O_6_	*Aspergillus nidulans*	NR	NA	[17]
neoaustin (**46**)	C_25_H_30_O_6_	*Penicillium* sp. MG-11	soil	NA	[36]
neosuatinone (**47**)	C_25_H_30_O_7_	*Aspergillus nidulans*	NR	NA	[17]
ED-2 (**48**)	C_25_H_30_O_8_	*Emericella nidulans* var. *dentata*	NR	NA	[6]
ED-2H (**49**)	C_25_H_32_O_8_	*Emericella nidulans* var. *dentata*	NR	NA	[6]
11*β*-acetoxyisoaustinone (**50**)	C_27_H_32_O_9_	*Pestalotiopsis* sp. PSU-ES194	the seagrass *Enhalus acoroides*	NA	[37]
		*Penicillium* sp.	*Dysosma versipellis*	NA	[38]
brasilianoid G (**51**)	C_25_H_28_O_7_	*Penicillium brasilianum* WZXY-m122-9	sponge	NA	[1]
brasilianoid H (**52**)	C_25_H_30_O_7_	*Penicillium brasilianum* WZXY-m122-9	sponge	NA	[1]
brasilianoid I (**53**)	C_25_H_28_O_8_	*Penicillium brasilianum* WZXY-m122-9	sponge	NA	[1]
brasilianoid L (**54**)	C_27_H_32_O_8_	*Penicillium brasilianum* WZXY-m122-9	sponge	cytotoxicity (RAW264.1, IC_50_ = 84.67 μg/mL; IEC-6, IC_50_ = 2.52 μg/mL; A549, IC_50_ = 180.5 μg/mL)	[1]
asperaustin A (**55**)	C_27_H_32_O_8_	*Aspergillus* sp.	the brown alga *Saccharina ichorioides* f. *sachalinensis*	NA	[2]
asperaustin B (**56**)	C_27_H_32_O_9_	*Aspergillus* sp.	the brown alga *Saccharina ichorioides* f. *sachalinensis*	NA	[2]
asperaustin C (**57**)	C_26_H_34_O_7_	*Aspergillus* sp.	the brown alga *Saccharina ichorioides* f. *sachalin ensis*	NA	[2]
6-hydroxyisoaustinone (**58**)	C_25_H_30_O_7_	*Penicillium* sp. GDGJ-285	the traditional Chinese medicinal plant *Sophora tonkinensis*	NA	[39]
6-ketoisoaustinone (**59**)	C_25_H_28_O_9_	*Penicillium* sp. GDGJ-285	the traditional Chinese medicinal plant *Sophora tonkinensis*	NA	[39]
penicianstinoid E (**60**)	C_27_H_32_O_10_	*Penicillium* sp. TGM112	the mangrove *Brguiera sexangula* var. *rhynchopetala*	inhibitory activity against newly hatched larvae of *Helicoverpa armigera* Hubner (IC_50_ = 200 μg/mL)	[22]
asperanstinoid A (**60**)		*Aspergillus calidoustus*	Dianchi Lake	NA	[24]
dhilirolide D (**61**)	C_25_H_30_O_7_	*Penicillium purpurogenum*	*Averrhoa bilimbi* fruit	NA	[13]
dhilirolide E (**62**)	C_25_H_30_O_7_	*Penicillium purpurogenum*	*Averrhoa bilimbi* fruit	NA	[12]
dhilirolide K (**63**)	C_25_H_28_O_8_	*Penicillium purpurogenum*	*Averrhoa bilimbi* fruit	NA	[12]
dhilirolide M (**64**)	C_25_H_28_O_7_	*Penicillium purpurogenum*	*Averrhoa bilimbi* fruit	NA	[12]
brasilianoid A (**65**)	C_26_H_34_O_8_	*Penicillium brasilianum* WZXY-m122-9	sponge	significantly stimulated the expression of filaggrin and caspase-14 in HaCaT cells	[34]
brasilianoid B (**66**)	C_25_H_32_O_6_	*Penicillium brasilianum* WZXY-m122-9	sponge	inhibitory effects on NO production (IC_50_ = 37.69 ± 5.25 μM)	[34]
brasilianoid C (**67**)	C_25_H_32_O_6_	*Penicillium brasilianum* WZXY-m122-9	sponge	inhibitory effects on NO production (IC_50_ = 33.76 ± 3.13 μM)	[34]
preaustinoid E (**68**)	C_25_H_32_O_6_	*Penicillium* sp. FCH061	sediment samples	NA	[40]
preaustinoid F (**69**)	C_25_H_32_O_6_	*Penicillium* sp. FCH061	sediment samples	NA	[40]
asperaustin C (**70**)	C_25_H_32_O_6_	*Aspergillus* sp.	the brown alga *Saccharina cichorioides* f. *sachalinensis*	NA	[2]
brasilianoid K (**71**)	C_25_H_32_O_7_	*Penicillium brasilianum* WZXY-m122-9	sponge	NA	[1]
dehydroaustin (**72**)	C_27_H_30_O_9_	*Aspergillum variecolor*	NR	growth inhibitory activity against the third-instar larvae of *Aedes aegypti* (LC_50_ = 2.9 ppm)	[7]
dehydroaustinol (**73**)	C_25_H_28_O_8_	*Penicillium* sp. MG-11	soil	NA	[36]
acetoxydehydroaustin (**74**)	C_29_H_32_O_11_	*Penicillium* sp. MG-11	soil	antimicrobial activity (*Escherichia coli*: 250 μg/mL); growth inhibitory activity against the third-instar larvae of *Aedes aegypti* (LC_50_ = 7.3 ppm)	[36]
ED-1 (**75**)	C_25_H_28_O_8_	*Emericella dentata*	NR	NA	[41]
ED-1H (**76**)	C_25_H_30_O_8_	*Emericella dentata*	NR	NA	[41]
acetoxydehydroaustin B′ (**77**)	C_29_H_32_O_11_	*Aspergillus* sp. 085241B	the Shankou Mangrove National Nature Reserve	NA	[42]
1,2-dihydro-acetoxydehydroaustin B′ (**78**)	C_29_H_34_O_11_	*Aspergillus* sp. 085241B	the Shankou Mangrove National Nature Reserve	NA	[42]
PF1364 (**79**)	C_31_H_36_O_13_	*Aspergillus* sp. PF1364	NR	control of pest and insect, such as the greenhouse whitefly	[43]
2-hydroacetoxydehydroaustin (**80**)	C_29_H_34_O_12_	*Aspergillus* sp. 16-5c	*Sonneratia apetala*	NA	[44]
1,2-dehydro-terredehydroaustin (**81**)	C_32_H_38_O_11_	*Aspergillus terreus* H010	*Kandelia obovata*	inhibitory effects on NO production (IC_50_ = 42.3 μM)	[45]
furanoaustinol (**82**)	C_25_H_30_O_9_	*Penicillium* sp. SF-5497	sea sand	inhibited the activity of protein tyrosine phosphatase 1B in a dose-dependent manner (IC_50_ = 77.2 μM)	[8]
7-acetoxydehydroaustinol (**83**)	C_27_H_30_O_10_	*Penicillium* sp. SF-5497	sea sand	inhibitory effects on NO production (IC_50_ = 61.0 μM)	[8]
asperaustin A (**84**)	C_27_H_32_O_8_	*Aspergillus* sp.	the brown alga *Saccharina cichorioides* f. *sachalinensis*	NA	[2]
asperaustin B (**85**)	C_27_H_32_O_9_	*Aspergillus* sp.	the brown alga *Saccharina cichorioides* f. *sachalinensis*	NA	[2]
brasilianoid J (**86**)	C_27_H_30_O_10_	*Penicillium brasilianum* WZXY-m122-9	sponge	NA	[1]
penicianstinoid A (**87**)	C_31_H_36_O_12_	*Penicillium* sp. TGM112	*Bruguiera sexangula* var. *rhynchopetala*	inhibition activity against newly hatched larvae of *Helicoverpa armigera* Hubner (IC_50_ = 200 μg/mL); insecticidal activity against *C. elegans* (EC_50_ = 9.4 ± 1.0 μg/mL)	[15]
penicianstinoid B (**88**)	C_25_H_28_O_9_	*Penicillium* sp. TGM112	*Bruguiera sexangula* var. *rhynchopetala*	inhibition activity against newly hatched larvae of *Helicoverpa armigera Hubner* (IC_50_ = 200 μg/mL); insecticidal activity against *C. elegans* (EC_50_ = 9.9 ± 0 μg/mL)	[15]
7-hydroxydehydroaustin (**89**)	C_27_H_30_O_10_	*Pestalotiopsis* sp. PSU-ES194	the seagrass *Enhalus acoroides*	NA	[37]
austinone (**90**)	C_32_H_40_O_13_	*Penicillium* sp. Y-5-2	NR	NA	[46]
ustusaustin A (**91**)	C_34_H_36_O_11_	*Aspergillus* ustus TK-5	the marine ascidian *Pyuramomus*	neuraminidase inhibitory activity (IC_50_ = 5.28 μM)	[47]
asperanstinoid B (**92**)	C_31_H_36_O_13_	*Aspergillus calidoustus*	Dianchi Lake	NA	[24]
asperanstinoid C (**93**)	C_32_H_38_O_12_	*Aspergillus calidoustus*	Dianchi Lake	NA	[24]
asperanstinoid D (**94**)	C_27_H_30_O_9_	*Aspergillus calidoustus*	Dianchi Lake	NA	[24]
peniclactone A (**95**)	C_26_H_28_O_9_	*Penicillium* sp. GDGJ-285	the traditional Chinese medicinal plant *Sophora tonkinensis*	NA	[39]
peniclactone B (**96**)	C_25_H_28_O_8_	*Penicillium* sp. GDGJ-285	the traditional Chinese medicinal plant *Sophora tonkinensis*	NA	[39]
peniclactone C (**97**)	C_26_H_28_O_8_	*Penicillium* sp. GDGJ-285	the traditional Chinese medicinal plant *Sophora tonkinensis*	inhibitory effects on NO production (IC_50_ = 39.03 μM)	[39]
dhilirolide L (**98**)	C_25_H_28_O_7_	*Penicillium purpurogenum*	*Averrhoa bilimbi* fruit	against the cabbage looper *Trichoplusia ni* (DC_50_ = 5.9 μg/cm^2^)	[12]
dhilirolide N (**99**)	C_25_H_26_O_8_	*Penicillium purpurogenum*	*Averrhoa bilimbi* fruit	NA	[12]
brasilianoid F (**100**)	C_25_H_32_O_7_	*Penicillium brasilianum* WZXY-m122-9	sponge	NA	[34]
dhilirolide A (**101**)	C_25_H_28_O_9_	*Penicillium purpurogenum*	*Averrhoa bilimbi* fruit	NA	[13]
dhilirolide B (**102**)	C_25_H_28_O_8_	*Penicillium purpurogenum*	*Averrhoa bilimbi* fruit	NA	[13]
dhilirolide C (**103**)	C_25_H_28_O_8_	*Penicillium purpurogenum*	*Averrhoa bilimbi* fruit	NA	[13]
dhilirolide J (**104**)	C_25_H_28_O_7_	*Penicillium purpurogenum*	*Averrhoa bilimbi* fruit	NA	[12]

^a^ Not reported. ^b^ No activity reported in the reference.

## Data Availability

Not applicable.
